# The role of math anxiety in U.S. adults’ ability to metacognitively monitor their fraction arithmetic performance

**DOI:** 10.3389/fpsyg.2026.1821523

**Published:** 2026-05-07

**Authors:** Samuel J. Pearl, Clarissa A. Thompson, Karrie E. Godwin, Jennifer M. Taber, Charles J. Fitzsimmons

**Affiliations:** 1 Department of Psychology, University of Kentucky, Lexington, KY, United States; 2 Department of Psychological and Brain Sciences, University of North Florida, Jacksonville, FL, United States; 3 Department of Psychological Sciences, Kent State University, Kent, OH, United States; 4 Department of Psychology, University of Maryland, Baltimore County, Baltimore, MD, United States

**Keywords:** confidence judgments, fraction arithmetic, individual differences, math anxiety, metacognitive monitoring, numerical cognition

## Abstract

Metacognitive monitoring, the awareness of one’s own knowledge, can inform control decisions like help-seeking. We examined whether and why math anxiety was related to metacognitive monitoring during fraction arithmetic. A combined sample of U.S. adults (*N* = 685) completed a fraction arithmetic task and judged how many questions they thought they would correctly answer prior to the task and how many questions they thought they correctly answered after the task. They also reported their math anxiety and math self-concept and completed a number-line estimation task. Adults tended to be underconfident in their performance, but their judgments were more accurate after compared to before the task. Additionally, adults with higher math anxiety had less accurate monitoring before and after completing the arithmetic task even when controlling for prior fraction knowledge. These findings suggest that math-anxious adults may struggle to accurately predict and reflect on their performance.

## Introduction

As a student practices math problems to prepare for a test, they may metacognitively judge whether they answered enough questions correctly to reach a desired level of performance (i.e., metacognitively monitor their performance; Nelson and Fyfe, 2019; Nelson, 1990; Rivers et al., 2019). These metacognitive judgments are thought to be informed by a variety of inferential cues such as past experiences with similar problem types, perceived difficulty while solving the problems, or beliefs about one’s own ability (Brunswik, 1955; Efklides, 2006; Koriat, 1997; Scheibe et al., 2022). The degree to which one’s judgments correspond to actual performance (i.e., monitoring accuracy) is important because metacognitive judgments can help guide control decisions such as asking for help or deciding to continue to study (Dunlosky and Thiede, 1998; Fitzsimmons and Thompson, 2023; Fyfe et al., 2022; Metcalfe and Finn, 2013; Nelson and Fyfe, 2019; Undorf et al., 2021). However, there are individual differences both in how accurate people are at monitoring their performance and the extent to which metacognitive judgments predict control behaviors like restudy choices and help seeking (Morehead and Dunlosky, 2020; Morehead et al., 2017; Peng and Tullis, 2020). Thus, it is important to understand how people judge their performance and the individual differences related to how well their judgments align with their performance.

We investigated the accuracy of adults’ judgments of their math performance when made prospectively (i.e., before a math task) and retrospectively (i.e., after a math task), and how individual differences in math anxiety, a feeling of tension, apprehension, or fear about math (Ashcraft, 2002), related to the accuracy of these judgments. We tested the hypothesis that math anxiety would be related to *more* accurate prospective monitoring, due in part to theoretical predictions that math anxiety may reflect self-awareness of one’s lower math ability. Below, we outline what metacognitive monitoring is, the cues that inform monitoring judgments, and the basis of our hypothesis that math anxiety may be related to more accurate prospective metacognitive monitoring.

### How do people monitor their performance?

According to inferential judgment models (i.e., Brunswik, 1955) such as the cue-utilization framework (Koriat, 1997), people judge their performance based on information related to the task (i.e., inferential cues), and judgments are accurate when the cues used to make judgments are predictive of performance. Koriat’s (1997) cue-utilization framework indicates that cues can be information-based, such as one’s perceived ability (Händel et al., 2020), the number of problems to be solved (Desender and Sasanguie, 2021), or the amount of time a person spent studying for an assessment (Koriat et al., 2008). Cues can also be experience based, such as item familiarity (Fitzsimmons and Thompson, 2022, 2023; Fitzsimmons et al., 2020; Koriat and Levy-Sadot, 2001; Metcalfe et al., 1993; Reder and Ritter, 1992), perceived task difficulty (e.g., Tiffin-Richards et al., 2022), or processing speed and reaction time (e.g., Ackerman and Koriat, 2011; Desender and Sasanguie, 2021; Koriat et al., 2008; Koriat et al., 2014). The information people use to judge their performance depends on whether they judge prospectively or retrospectively. Prospective judgments likely rely on information-based cues because people have not yet engaged with the task itself. However, when making a judgment after completing a task (i.e., a retrospective judgment), people can rely on additional information related to their experiences during the task. For example, Desender and Sasanguie (2021) found that perceived task difficulty predicted prospective judgments, but actual task performance predicted retrospective judgments. Given that experiences during the task provide additional cues about one’s own performance, people’s judgments tend to be more accurate retrospectively compared to prospectively, an effect coined the *postdiction superiority effect* (e.g., Benjamin, 2003; Pierce and Smith, 2001).

The postdiction superiority effect sometimes occurs in math (Boekaerts and Rozendaal, 2010; Lingel et al., 2019). For example, Lingel et al. (2019) found that students were more accurate at judging their performance after compared to before an algebraic and word problem task. Additionally, they found that students with lower math achievement scores had greater prospective overconfidence compared to those with higher math achievement scores, but there was no difference in overconfidence by achievement level for retrospective judgments. This finding suggests that the postdiction superiority effect may vary by people’s prior knowledge. However, it is unclear whether other individual differences like math anxiety are also related to the postdiction superiority effect.

### How does math anxiety relate to metacognitive monitoring?

Math anxiety is often negatively related to math performance (Barroso et al., 2021; Foley et al., 2017; Hembree, 1990; Ma, 1999; Sidney et al., 2019). The Disruption Account (Ashcraft and Kirk, 2001), the Reduced Competency Account (Maloney, 2016), and the Interpretation Account (Ramirez et al., 2018) of math anxiety all differ in the hypothesized causal direction between anxiety and performance. The Disruption Account suggests that worries about performance take up working memory resources and lead to lower-than-expected math performance (Ashcraft and Kirk, 2001). In other words, the Disruption Account suggests math anxiety causes low performance. However, both the Reduced Competency Account and the Interpretation Account suggest that poor math performance causes math anxiety (Maloney, 2016; Ramirez et al., 2018). The Interpretation Account differs in that it suggests that attribution of prior math performance (i.e., as due to ability or external factors) plays an important role in the development of math anxiety and explains why some people have low math performance and low math anxiety or high math performance and high math anxiety. In support of this view, students’ math self-concept but not actual ability predicted math anxiety one year later (Meece et al., 1990). We believe all three frameworks can inform hypotheses about the relation between math anxiety and metacognitive monitoring.

#### Reduced competency account

The Reduced Competency Account implies that math-anxious adults are aware of their poor math performance. That is, for one to develop math anxiety as a result of poor performance, they must be aware of their prior poor ability. Thus, we expected that those with higher math anxiety are aware that they have lower math ability in general and would therefore be more accurate at predicting their future math performance. Our prediction is based on evidence showing that adults in general tend to be *overconfident* in their math ability (e.g., Erickson and Heit, 2015; Lingel et al., 2019; Morsanyi et al., 2014). Thus, we expected people to be overconfident prospectively, but we expected that people with higher math anxiety would be less overconfident prospectively (i.e., more accurate in their judgments about how many arithmetic problems they would correctly answer).

In addition to *perceived* ability, math anxiety may relate to monitoring accuracy because of the relation between math anxiety and *actual* ability. For example, people with lower ability tend to be more overconfident in their performance, coined the *Dunning-Kruger Effect* (Kruger and Dunning, 1999). In math, the Dunning-Kruger Effect has been found for prospective but not retrospective monitoring judgments (Lingel et al., 2019). In line with The Reduced Competency account, if people develop math anxiety due to their poor math ability, this poor math ability may also explain math anxiety’s relation to metacognitive monitoring.

#### Interpretation account

The Interpretation Account suggests that the relation between math anxiety and prospective monitoring accuracy would be due, in part, to the relation between math anxiety and math self-concept. Math self-concept is a personal belief about one’s competence in math.^1^ Evidence suggests that math self-concept is related to math anxiety (Ahmed et al., 2012; Meece et al., 1990; Sokolowski et al., 2019), and people may develop math anxiety if they perceive their poor math performance to be reflective of their ability (i.e., a low math self-concept). Thus, we reasoned that the Interpretation Account would predict that controlling for math self-concept would attenuate or eliminate the relation between math anxiety and prospective monitoring accuracy based on the reasoning that math-anxious adults may reflect on their math self-concept when making monitoring judgments. In hindsight, we also realized that math self-concept and math anxiety should be highly related and have similar relations to metacognitive monitoring.

#### Disruption account

We reason that the Disruption Account makes predictions about ongoing or retrospective monitoring accuracy. If math anxiety disrupts working memory resources during math problem-solving, math-anxious individuals may be less able to identify and use cues related to their experiences during problem solving when making retrospective judgments. Thus, we believe that the Disruption Account would predict that higher math anxiety would be related to lower retrospective monitoring accuracy. Supporting this hypothesis, Bellon et al. (2021) found that children with higher math anxiety made less accurate retrospective monitoring judgments in an addition and multiplication task.

In contrast to our interpretation of the Disruption Account of math anxiety, adults also appear to be more sensitive to errors if they have higher math anxiety. For example, adults’ math anxiety was related to a greater error-related negativity (i.e., a neurological response to committing errors) in a numerical but not a non-numerical Stroop task (Suárez-Pellicioni et al., 2013). Suárez-Pellicioni et al. (2013) operationalized error-related negativity as a negative deflection in brainwave activity in the areas of the brain related to error sensitivity as measured with EEG. This sensitivity to errors may reflect a metacognitive process related to one’s affective response to committing errors. If this sensitivity to committing errors informs monitoring judgments, then higher math anxiety would be associated with more accurate retrospective monitoring. Thus, we did not have a directional hypothesis about the relation between math anxiety and retrospective monitoring accuracy. Instead, a negative relation between math anxiety and monitoring accuracy for retrospective judgments may indicate that those with math anxiety have disruptions in their processing during math tasks (i.e., supporting the Disruption Account), whereas a positive relation between math anxiety and monitoring accuracy may indicate that math-anxious individuals are more sensitive to errors (i.e., supporting the error-sensitivity hypothesis).

### Fraction arithmetic and whole number bias

We assessed adults’ ability to metacognitively monitor their fraction-arithmetic performance because fraction knowledge is important for academic (e.g., Bailey et al., 2012; Booth et al., 2014; Siegler et al., 2012; Viegut et al., 2023) and career success (e.g., Handel, 2016). Despite the importance of fraction knowledge, adults tend to have negative attitudes about math with fractions (e.g., Mielicki et al., 2021; Sidney et al., 2021) and struggle with fraction arithmetic (Godwin et al., 2023; Lortie-Forgues and Siegler, 2017; Siegler and Pyke, 2013).

One reason children and adults struggle with fractions is because they incorrectly apply whole-number strategies when reasoning about fractions, an error called whole number bias (i.e., Alibali and Sidney, 2015; Ni and Zhou, 2005). Whole number bias is prevalent among children, adults, math experts, and teachers, and can be seen in a variety of tasks such as comparison, estimation, ordering, density (i.e., how many numbers are between 1/2 and 1/4), and arithmetic (e.g., Depaepe et al., 2015; Obersteiner et al., 2013; Van Hoof et al., 2015).

Whole-number bias errors in fraction arithmetic have previously been related to math anxiety (e.g., González-Forte et al., 2022; Halme et al., 2024; Van Hoof et al., 2025). Recent work has found that students tend to report lower levels of state fraction anxiety when they make whole-number bias errors compared to other errors during fraction reasoning (Halme et al., 2024). As students get better at solving fraction arithmetic problems and overcome whole number bias, they report higher levels of state anxiety (Van Hoof et al., 2025).

Despite the link between fraction arithmetic performance and math anxiety, adults may be unaware of their fraction errors because of some of the misconceptions they have about fractions. For example, González-Forte et al. (2023) interviewed 7th-grade students about the strategies they used to previously solve fraction comparison problems and had them rate how sure they were in their reasoning. They found that students who made whole-number bias errors were relatively confident in their answers, but that students who were accurate were more confident than those who made errors. These prior studies suggest that some students may make errors, have low math anxiety, but have relatively high confidence. As students’ accuracy improves in solving fraction arithmetic, they may have higher confidence even when their math anxiety increases. However, no studies to our knowledge have examined whether trait math anxiety is related to adults’ metacognitive awareness of their fraction arithmetic performance.

### Current study

In the current study, we assessed whether and how math anxiety related to adults’ ability to metacognitively monitor their fraction arithmetic performance. During the fraction arithmetic task, adults provided prospective judgments about how many problems they would answer correctly overall and separately for each operation (i.e., addition, subtraction, multiplication, and division). They also provided retrospective confidence judgments after they solved each problem as well as about their overall accuracy and for each operation after completing all problems.

#### Hypotheses

We made four pre-registered hypotheses.^2^ We expected those with greater math anxiety to make more accurate prospective monitoring judgments (H1). We also pre-registered that we expected the relation between math anxiety and prospective monitoring accuracy to be attenuated or eliminated after controlling for math self-concept (H2). However, we could not directly test this hypothesis due to issues of multicollinearity between math anxiety and math self-concept. Finally, we explored whether the relation between math anxiety and prospective monitoring accuracy was attenuated or eliminated when controlling for prior math knowledge.

Although we did not have directional hypotheses about the relation between math anxiety and retrospective monitoring accuracy, we reasoned that if people with higher math anxiety are more sensitive to errors (Suárez-Pellicioni et al., 2013), and they use this error sensitivity to make judgments of their performance, then higher math anxiety should be associated with more accurate retrospective monitoring. However, those with higher math anxiety may disengage with math (Ashcraft and Kirk, 2001) or have disruptions in their working memory system (e.g., Ramirez et al., 2018), which would disrupt their ability to use experience-based cues to inform their retrospective judgments. Thus, in line with the Disruption Account, math anxiety may be unrelated or negatively related to retrospective monitoring accuracy.

We also expected participants’ judgments to be more accurate retrospectively compared to prospectively (i.e., a postdiction superiority effect; H3). And, we expected math anxiety to moderate the postdiction superiority effect such that the difference in monitoring accuracy for prospective and retrospective judgments would be smaller for those with higher relative to lower levels of math anxiety (H4).

#### Monitoring indices

We considered two different types of metacognitive monitoring: absolute accuracy and relative accuracy. Absolute accuracy reflects the precision of one’s judgments; that is, how closely the judgment matches performance (Dunlosky et al., 2016). Relative accuracy reflects how well participants’ judgments discriminate between accurate and inaccurate performance. Relative accuracy considers the ordered relation between judgments and performance such that higher relative accuracy reflects people having higher confidence when they are more accurate and lower confidence when they are less accurate. Although we would generally expect that results for absolute and relative accuracy would converge (Dunlosky and Thiede, 2013; Schraw et al., 2012), there is reason to believe that the psychological processes and the cues that one relies on to make judgments will vary by judgment time. For example, relative accuracy relies on one’s ability to discriminate between problems they answered correctly versus incorrectly, but absolute accuracy does not involve comparison between problems. And, it makes sense that people would use different cues to make predictions or postdictions about overall performance compared to judgments about how accurately they solve individual problems. Given differences in the underlying psychological processes involved in these measures of monitoring accuracy, it is plausible there may be different relations among individual differences and these measures of monitoring. We focused our hypotheses and analyses on aggregate level prospective and retrospective judgments, though we also report relative accuracy based on item-level judgments for completeness and transparency.

## Method

### Transparency and openness statement

The hypotheses in this study were pre-registered after data collection but prior to analyses (see text footnote 2). We describe each measure relevant to the hypotheses in the current study, though other measures were included as part of a larger study examining a different set of research questions. All data and analytic scripts are available on OSF (https://osf.io/kfs2h/overview). Note that we deviated from our initial pre-registered analysis plan by combining two samples rather than analyzing those data separately. Importantly, our conclusions were the same regardless of our analytic approach. We detail the differences in our initial analytic approach and our current manuscript in our Supplementary File.

### Participants

We recruited two samples of participants as part of a larger study investigating the role of self-affirmation and training on people’s math performance in a health domain. A prior publication used these data to examine people’s attention as they solved fraction arithmetic problems (Godwin et al., 2023). Here, we use the data to evaluate our questions about monitoring ability.

As part of a larger parent study, we recruited U.S. adults through an online platform, Prolific, in two separate samples (*N* = 420 & *N* = 355). Given that this was a secondary data analysis, the power analyses for recruitment were unrelated to the planned analyses in the present study. The sample sizes were determined based on a power analysis conducted in G*Power to detect a small-to-medium effect size (*f* = 0.15, alpha = 0.05, power = 80%) for an ANOVA with four groups in the first sample and an ANCOVA with 2 groups and one covariate in the second sample. As in Godwin et al. (2023), we excluded 41 participants in Sample 1 and 49 participants in Sample 2 for survey completion percentage or time, missing two attention checks, and/or responding with gibberish answers to open-ended questions. The final samples consisted of 379 participants in Sample 1 and 306 participants in Sample 2. Of the final sample who reported demographics, there were similar numbers of males and females in both samples, most participants had a bachelor’s degree or greater, identified as White, were employed, or had an income of $74,999 or less. The age of participants was not included in the demographic survey across both samples. We reproduce full demographic information in Table 1.

**Table 1:** Demographic information.

Demographic variable	*N*	%
Gender		
Male	328	47.9%
Female	333	48.6%
Nonbinary	16	2.3%
Other	1	0.1%
Did not report	7	1.0%
Education levels		
Less than high school graduate	7	1.0%
High school graduate/GED	98	14.3%
Some college or trade school	125	18.2%
Associate degree	61	8.9%
Bachelor’s degree	279	40.7%
Graduate degree	112	16.4%
Did not report	3	0.4%
Race/ethnicity		
White	498	74.7%
Black or African American	51	7.6%
American Indian or Alaska Native	0	0.0%
Asian	53	7.9%
Native Hawaiian or Pacific Islander	0	0.0%
Hispanic or Latino	32	4.8%
Two or more	25	3.7%
Other	1	0.1%
Did not report	7	1.0%
Employment		
Employed for wages	396	57.8%
Self-employed	94	13.7%
Out of work for more than a year	52	7.6%
Out of work for less than a year	25	3.6%
Homemaker	21	3.1%
Student	59	8.6%
Retired	21	3.1%
Did not report	17	2.5%
Household income level		
<$15,000	49	7.0%
$15,000 – $24,999	63	9.1%
$25,000 – $34,999	65	9.3%
$35,000 – $49,999	105	15.1%
$50,000 – $74,999	123	17.7%
$75,000 – $99,999	113	16.2%
$100,000 – $149,999	89	12.8%
$150,000 – $199,999	39	5.6%
≥$200,000	21	3.0%
Did not report	29	4.2%

### Procedure

Participants completed a series of math-related tasks online through the survey software Qualtrics. Participants completed a block of math tasks (i.e., number-line estimation and a fraction arithmetic task) and a block of math attitude tasks (i.e., Math Attitudes Questionnaire and a math anxiety questionnaire) in a counterbalanced order. Then, as a part of a larger study, we randomly assigned participants to read either a math or non-math message and evaluated whether participants perceived the math message as threatening. The current study focused only on the tasks administered prior to the manipulation, so we do not consider the effect of the manipulation here. A full list of measures included prior to the manipulation is included in Supplementary File.

### Measures

#### Prior fraction knowledge

In Sample 1, participants estimated the location of 10 fractions on a 0–1 number line and 10 fractions on a 0–5 number line (adapted from a subset of items in Fitzsimmons and Thompson, 2022). Participants estimated five magnitudes in the 0–1 range with half of the fractions having small components (e.g., 1/6) and the other half being equivalent in magnitude but having larger components (e.g., 12/72). In Sample 2, participants estimated the location of 10 fractions on a 0–5 number line. Here we only include performance on the 0–5 number-line estimation task as an index of prior fraction knowledge because this measure was the same for both samples. The list of stimuli are in Supplementary File. To measure performance, we calculated the percent absolute error (PAE, i.e., |(estimate – magnitude)|/numerical range * 100%) for each estimate and then calculated average PAE for each participant for the 0–5 number line estimates. We considered performance on the number-line task to be indicative of prior fraction knowledge because performance on number-line tasks is strongly related to and predictive of math achievement (Fazio et al., 2014; Siegler et al., 2011; Thompson et al., 2023; Ünal et al., 2024).

#### Fraction arithmetic

Participants completed six fraction arithmetic problems for each operation (i.e., addition, subtraction, multiplication, and division; Godwin et al., 2023; Siegler and Pyke, 2013) for a total of 24 problems. The fraction arithmetic problems were blocked by operation. For each operation, three problems had equal denominators (e.g., 3/5 + 4/5) and three had different denominators (e.g., 9/36–27/45). To report their answer, participants entered the numerator and denominator of the fraction into two text boxes. We used the same coding scheme for accuracy as reported in Godwin et al. (2023). Non-responses were coded as incorrect. We considered an accurate response as one in which the entered fraction was equivalent to the equivalent decimal correct answer, regardless of whether the fraction was reduced, nonreduced, a mixed fraction, or included a decimal value. We calculated mean accuracy for all 24 problems as well as by operation type. Reliability was *α* = 0.94.

#### Aggregate metacognitive judgments

Before completing the fraction arithmetic task, participants estimated how many problems they expected to correctly answer overall (out of 24), and then how many problems they expected to correctly answer by operation (out of 6 per operation). Participants were told “Next, you’ll complete some fraction arithmetic problems. You’ll complete 24 total problems: 6 fraction addition, 6 fraction subtraction, 6 fraction multiplication, and 6 fraction division” and then asked “How many questions do you think you’ll answer correctly in total out of 24? Please type a number between 0 and 24.” Then, for each operation, they were asked how many questions they would correctly answer from 0 to 6. After completing the fraction arithmetic task, participants estimated how many problems they thought they correctly answered overall (out of 24) and by operation. They answered the question: “You just completed some fraction arithmetic problems. You completed 24 total problems: 6 fraction addition, 6 fraction subtraction, 6 fraction multiplication, and 6 fraction division.” and then asked “How many questions do you think you answered correctly in total out of 24? Please type a number between 0 and 24.” Then, for each operation, they were asked how many questions they correctly answered from 0 to 6.

We calculated bias and absolute bias (Dunlosky et al., 2016; Rivers et al., 2019). Bias was calculated as the signed difference between the metacognitive judgment and performance (i.e., judgment minus performance). Thus, positive values of bias reflect overconfidence and negative values of bias reflect underconfidence. We calculated absolute bias as the absolute value of the difference between the metacognitive judgment and performance (i.e., the absolute value of judgment minus performance; Pressley et al., 1984). Larger values indicate less accurate judgments. For both measures, a value closer to 0 reflects more accurate judgments. We calculated measures of bias and absolute bias based on participants’ overall judgment (i.e., total number of questions correct) rather than the operation-level judgments because we did not have specific hypotheses about operation-level monitoring accuracy. Because there are more items in total than for each operation, overall judgments also allow for more variability.

#### Item-level metacognitive judgments

After each fraction arithmetic item, participants reported their confidence on a continuous scale from 0 (*I am not confident at all*) to 100 (*I am totally confident*) and made a dichotomous rating (i.e., “Do you think you answered this question correctly?”). We calculated the within-person Gamma correlation between judgments and accuracy across all items (Fitzsimmons et al., 2023; Nelson, 1984; Wall et al., 2016). We focus on the relation between item-level continuous judgments and performance, though results were consistent when analyzed with continuous or dichotomous gamma.

#### Math anxiety

Participants completed a seven-item math anxiety questionnaire (Mielicki et al., 2023; Thompson et al., 2021). They reported how math-anxious they were in general, about whole numbers, fractions, and each fraction operation (i.e., addition, subtraction, multiplication, division) on a scale from 1 (*not anxious*) to 10 (*very anxious*). Math anxiety was operationalized as the mean across all items. Reliability in our sample was *α* = 0.95.

#### Math self-concept

Participants completed a shortened Math Attitudes Questionnaire (MAQ; Mielicki et al., 2021; Sidney et al., 2021). The MAQ has four subscales that focus on self-perceived ability, preferences, perceived frequency of use, and perceived importance of math. Within the ability section of the shortened MAQ, participants reported how good they are at math, how good they are at thinking about how big fractions are, and how good they are at solving fraction problems on a scale from 1 (*very bad*) to 6 (*very good*). We operationalized math self-concept as the mean score from the three items in the ability section. The reliability in our sample was *α* = 0.92.

## Results

### Analytic overview

First, we report general descriptive statistics and correlations (Tables 2, 3). Then, we report a hierarchical linear regression in which we predict metacognitive accuracy (as bias or absolute bias) from math anxiety and prior fraction knowledge to evaluate whether math anxiety still predicts monitoring when controlling for prior ability. We then report a series of repeated-measures general linear models in which we predict metacognitive accuracy (as bias or absolute bias) from judgment time (prospective and retrospective). Finally, we report whether math anxiety moderated the effect of judgment time on monitoring accuracy.

**Table 2:** Descriptive statistics.

Variables (possible range) [*N*]	Mean (SD) [min – max]
Fraction arithmetic accuracy (0–100%) [685]	67.42% (6.79%) [0–24]
Prospective confidence judgment (0–100%) [684]	54.91% (29.58%) [0–24]
Retrospective confidence judgment (0–100%) [684]	56.88% (32.08%) [0–24]
Prospective bias (−24 to 24) [684]	−3.00 (6.69)^*^ [−22–22]
Retrospective bias (−24 to 24) [684]	−2.52 (5.06)^*^ [−19–22]
Prospective absolute bias (0–24) [684]	5.73 (4.57)^*^ [0–22]
Retrospective absolute bias (0–24) [684]	4.19 (3.79)^*^ [0–22]
Continuous item-level gamma (−1 to 1) [545]	0.43 (0.52)^*^ [−1–1]
Math anxiety (1–10) [685]	5.37 (2.57) [1–10]
Math self-concept (1–6) [685]	3.64 (1.30) [1–6]
Percent absolute error (0–100%) [685]	12.82% (11.1%) [1.09–51.3%]

* Indicates that the mean is significantly different from zero for the measures of monitoring accuracy (i.e., prospective/retrospective bias/absolute bias and continuous gamma). We used a one-tailed test for absolute bias because all values are positive.

**Table 3:** Correlations among variables.

Variable names	1	2	3	4	5	6	7	8	9	10
1. Fraction arithmetic accuracy										
2. Prospective confidence judgment	0.54**									
3. Retrospective confidence judgment	0.76**	0.76**								
4. Prospective bias	−0.45**	0.52**	0.03							
5. Retrospective bias	−0.18**	0.43**	0.50**	0.64**						
6. Prospective absolute bias	−0.01	−0.35**	−0.18**	−0.37**	−0.26**					
7. Retrospective absolute bias	−0.15**	−0.38**	−0.47**	−0.25**	−0.52**	0.55**				
8. Continuous item-level gamma	−0.17**	−0.14**	−0.20**	0.01	−0.07	< 0.01	0.04			
9. Math anxiety	−0.50**	−0.62**	−0.58**	−0.15**	−0.21**	0.21**	0.28**	0.10*		
10. Math self-concept	0.56**	0.73**	0.68**	0.21**	0.29**	−0.20**	−0.25**	−0.12**	−0.73**	
11. Percent absolute error (number-line estimation)	−0.56**	−0.42**	−0.50**	0.13**	−0.01	0.10*	0.18**	−0.06	0.45**	−0.45**

Fraction arithmetic accuracy was reported in Godwin et al. (2023). Prospective = before the task. Retrospective = after the task. Aggregate judgments are judgments of overall performance (e.g., total number correct). Bias reflects the signed difference between judgments and performance; positive bias reflects overconfidence and negative values reflect underconfidence. Absolute bias is the absolute value of the difference between judgments and performance. Gamma is an ordinal measure of relative monitoring accuracy. Percent absolute error is a measure of prior fraction knowledge; lower values indicate greater knowledge.

*p < 0.05. **p < 0.01.

Participants were more accurate (*M* = 67.42%) than they predicted they would be prospectively (*M* = 54.91%) or retrospectively (*M* = 56.88%). Thus, adults were underconfident in their fraction arithmetic performance before and after the task, inconsistent with previous work on monitoring in math and other domains (Erickson and Heit, 2015; Lingel et al., 2019; Morsanyi et al., 2014).

### Is math anxiety related to monitoring accuracy?

Contrary to our hypothesis (H1), math anxiety was related to greater underconfidence (*r* = −0.15) and less accurate absolute bias (*r* = 0.21) for prospective judgments (see Table 3). Those with more math anxiety were less accurate at predicting how many fraction arithmetic problems they would solve correctly.

We explored whether math anxiety continued to predict prospective monitoring accuracy after controlling for previous fraction knowledge. As can be seen in Table 4, higher levels of math anxiety continued to significantly predict less accurate prospective monitoring accuracy when controlling for previous fraction knowledge. Thus, math anxiety is a unique predictor of prospective monitoring accuracy beyond prior knowledge.

**Table 4:** Hierarchical linear regression for math anxiety predicting prospective monitoring accuracy.

Predictor	Prospective bias (over/under confidence)			Prospective absolute bias		
	*b* (SE)	*β*	[*β* 95% CI]	*b* (SE)	*β*	[*β* 95% CI]
Constant	−1.30 (0.58)*	–	–	3.74 (0.40)***	–	–
Math anxiety	−0.66 (0.11)***	−0.25***	[−0.47, −0.04]	0.37 (0.07)***	0.20***	[0.06, 0.35]
Percent absolute error	0.15 (0.02)***	0.24***	[0.19, 0.29]	< 0.01 (0.02)	<0.01	[−0.03, 0.04]
	*F*(2, 681) = 24.63***, adjusted *R*^2^ = 0.06			*F*(2, 681) = 15.46***, adjusted *R*^2^ = 0.04		

Lower values of percent absolute error indicate greater fraction knowledge. *p < 0.05, **p < 0.01, ***p < 0.001.

In line with the Interpretation Account, we hypothesized that math-anxious adults might reflect on their math self-concept when making monitoring judgments (H2). If so, we would expect strong relations between math self-concept and math anxiety, and similar relations between these variables and monitoring accuracy. As expected, math anxiety and math self-concept were highly related (*r* = −0.73), and the correlations between self-concept and monitoring accuracy were very similar to those between math anxiety and monitoring accuracy (see Table 3). These correlations suggest there may be considerable overlap between math anxiety and math self-concept.

We also explored the relation between math anxiety and retrospective aggregate- and item-level monitoring accuracy. Similar to our results on prospective monitoring, adults with higher math anxiety were more underconfident (*r* = −0.21) and had less accurate absolute bias (*r* = 0.28) when judging their performance retrospectively. Although higher levels of math anxiety were related to less accurate monitoring before and after the arithmetic task, math anxiety was related to more accurate relative monitoring accuracy (i.e., measured as gamma correlations; *r* = 0.10). That is, adults with higher levels of math anxiety were better able to distinguish between items they answered correctly and incorrectly. The positive relation between math anxiety and relative monitoring accuracy is consistent with the hypothesis that math-anxious adults might be more sensitive to errors they make as they solve arithmetic problems. The difference in the relation between math anxiety and item-level relative accuracy and aggregate level prospective and retrospective accuracy suggests that adults likely use different cues when monitoring ongoing performance compared to making aggregate-level predictions or postdictions. However, the relation between continuous gamma correlations and math anxiety was small and prior work has found no significant relation between math anxiety and item-level monitoring (e.g., Fitzsimmons and Thompson, 2022). Thus, future research should continue to explore the relation between math anxiety and relative monitoring accuracy.

### Is there a postdiction superiority effect and does it depend on one’s math anxiety?

We found evidence of a postdiction superiority effect (H3) for bias (i.e., the signed difference between a judgment and performance) and absolute bias (i.e., the absolute difference between a judgment and performance). A repeated-measures general-linear model indicated that adults were less underconfident when judging their fraction arithmetic retrospectively (*M* = −2.51, *SD* = 0.19) compared to prospectively (*M* = −2.98, *SD* = 0.26), *F*(1, 682) = 5.74, *p* = 0.017, *η*_p_^2^ = 0.01. Similarly, a repeated-measures general-linear model indicated that adults’ judgments were more aligned with their performance when made retrospectively (*M* = 4.18, *SD* = 0.14) compared to prospectively (*M* = 5.72, *SD* = 0.17), *F*(1, 682) = 99.69, *p* < 0.001, *η*_p_^2^ = 0.13.

To examine whether the difference in monitoring accuracy by time depended on math anxiety (H4), we added mean-centered math anxiety to the previous models. We found no significant interaction between math anxiety and time (i.e., judgments before/after completing the math problem) for bias, *F*(1, 681) = 0.13, *p* = 0.717, *η*_p_^2^ < 0.001, or absolute bias, *F*(1, 681) = 0.50, *p* = 0.480, *η*_p_^2^ < 0.001.

## Discussion

Metacognitive monitoring is important because it can relate to self-regulatory control decisions like asking for help or choosing to restudy (Dunlosky and Thiede, 1998; Fitzsimmons and Thompson, 2023; Fyfe et al., 2022; Metcalfe and Finn, 2013; Nelson and Fyfe, 2019; Undorf et al., 2021). To our knowledge, this is the first study to investigate how highly educated adults monitor their arithmetic performance and how math anxiety relates to monitoring during fraction arithmetic. We found that adults were less accurate in making overall prospective and retrospective judgments if they had higher math anxiety, but were better able to distinguish between items they answered correctly and incorrectly when judging ongoing performance. Adults’ judgments were more aligned with their performance when made retrospectively compared to prospectively (i.e., the postdiction superiority effect), but this effect was not moderated by math anxiety.

Although students tend to be overconfident in their math ability (Erickson and Heit, 2015; Lingel et al., 2019; Morsanyi et al., 2014), adults in the current study were underconfident when judging their fraction arithmetic performance. One possibility is that people may be underconfident in their fraction arithmetic performance because they generally have less favorable attitudes about fractions compared to math with whole numbers or math in general (Mielicki et al., 2021; Sidney et al., 2021). Thus, adults might be overconfident in their ability to complete other types of math (e.g., whole number arithmetic or decimal arithmetic). Alternatively, participants’ high level of education may have contributed to their underconfidence (i.e., over 53% of the sample had a bachelor’s degree or higher). Prior research has found that as people become more knowledgeable, they often become more accurate at judging their performance and sometimes even become *underconfident* (see Kruger and Dunning, 1999). For example, Ghazal et al. (2014) found that higher levels of numeracy were related to less overconfidence (i.e., more accurate monitoring) on a medical comprehension task.

In contrast to our first hypothesis, a key finding in the current study was that math anxiety related to less accurate aggregate-level monitoring accuracy. Our initial hypothesis about math anxiety relating to more accurate prospective monitoring was based on our expectation for adults to be overconfident in their fraction arithmetic ability, and for more math-anxious adults to be less overconfident. However, our data suggest that instead of being more aware of their true ability to solve problems, adults with higher math anxiety perceive their ability to be lower and judge that they will solve or did solve fewer problems correctly (see Table 4). Thus, future research may investigate the relation between math anxiety and monitoring in tasks for which adults are generally overconfident in their ability, such as algebra or word problems (e.g., Lingel et al., 2019).

Although our results are inconsistent with our hypothesis, they may help inform how math-anxious adults perceive their math ability. In particular, our study suggests that math-anxious adults may perceive their ability to be lower than their actual performance. This is concerning because, according to motivational research (Chong et al., 2016), math-anxious adults may reflect on their low confidence when making cost–benefit analyses, leading to less optimal decisions. For example, math-anxious individuals who judge their ability as lower than their true performance (i.e., underconfidence) may perceive that it will require too much effort to complete a task, leading them to select an easier task or not complete the task at all (e.g., choosing to study a topic they know more about compared to studying a topic they know less about). Consistent with this reasoning, Choe et al. (2019) found that math-anxious adults chose to solve an easier versus harder arithmetic problem even though doing so meant forgoing a greater reward.

Another reason math anxiety may have been negatively related to monitoring accuracy and confidence judgments could be due to whole-number bias (Alibali and Sidney, 2015; Ni and Zhou, 2005). It is likely that some of the adults in our study made errors based on using whole-number strategies to solve the problem. When students make whole-number bias errors, they tend to report lower levels of state fraction anxiety than when they make other types of errors, possibly representing a lack of awareness about their performance (e.g., Halme et al., 2024; Van Hoof et al., 2025). Additionally, some work suggests that students have relatively high confidence in these errors (e.g., around 79% confident; González-Forte et al., 2023). Thus, the negative relation between math anxiety and confidence could be partially explained by a subset of low-anxious participants who commit whole-number bias errors while remaining relatively confident. However, Halme et al. (2024) did not find differences in trait-level math anxiety, which was measured in the current study, for those who committed whole-number bias errors compared to other types of errors during fraction arithmetic. Future work could investigate how whole-number bias errors relate to monitoring and math anxiety.

Our results for retrospective monitoring accuracy suggest people rely on different psychological processes to engage in absolute compared to relative monitoring accuracy. Math anxiety was related to less accurate retrospective aggregate monitoring but was related to more accurate relative monitoring accuracy (i.e., distinguishing between arithmetic items they answered correctly and incorrectly). Neurological work suggests math-anxious adults might have more accurate relative monitoring because they may be more sensitive to committing errors (Suárez-Pellicioni et al., 2013), and this sensitivity may be important for in-the-moment monitoring. However, this effect was small, and prior work has found no significant relation between math anxiety and relative monitoring accuracy (Fitzsimmons and Thompson, 2022).

Contrary to research on neurological sensitivity to errors (e.g., Suárez-Pellicioni et al., 2013), the Disruption Account suggests that math-anxious adults may have less accurate retrospective monitoring judgments because math anxiety might preoccupy working memory resources and disrupt adults’ ability to pick up on experience-based cues during the task that would be informative when making retrospective judgments (Ashcraft and Kirk, 2001). Consistent with this reasoning, math-anxious adults had less accurate aggregate monitoring accuracy after the fraction arithmetic task. However, previous work has found mixed evidence on the role of working memory in metacognitive monitoring. For example, Bryce et al. (2023) found that adults were less accurate at monitoring when there was a greater demand on working memory on a visuospatial complex span task and an updating task (see Bryce et al., 2023 for a detailed description of the tasks). In contrast, Roebers et al. (2009) found that children’s working memory capacity was not related to monitoring ability on a science-related task. However, neither our own, nor prior work, assessed both math anxiety and working memory to better evaluate whether math anxiety is related to monitoring accuracy because of disruptions in working memory. Thus, the role of working memory in math anxiety’s relation to monitoring should be further explored.

### Limitations

The current study has some limitations. First, our sample included generally well-educated adults, which may be why adults were underconfident in the current study, in contrast to work where students tend to be overconfident in math (Erickson and Heit, 2015; Lingel et al., 2019; Morsanyi et al., 2014). Future work could investigate whether these relations replicate in a sample of adults with lower educational attainment, those still in college, or in other types of arithmetic.

Additionally, although our measure of math self-concept was adopted from the self-perceived ability subscale of the Math Attitudes Questionnaire (Mielicki et al., 2021; Sidney et al., 2021), this subscale has not been validated against other measures of math self-concept. This measure of math self-concept was strongly correlated with math anxiety, but other work has found a moderately strong correlation between the two variables (*r*’s near 0.48) in Dutch children (Ahmed et al., 2012). Future work could include multiple measures of math self-concept and math anxiety to evaluate the measurement characteristics of these scales.

### Implications and future directions

If math anxiety disrupts people’s ability to monitor their performance, this might have implications for how they engage in metacognitive control decisions, such as asking for help or restudying topics for an upcoming test. For example, if a math-anxious student is unconfident in their ability to do well on an upcoming test, then according to some self-regulated theories, they may be likely to ask for help (e.g., Dunlosky and Thiede, 1998). However, theories of math avoidance in math anxiety might suggest that math-anxious individuals may not ask for help even if they know they are performing poorly (see Ramirez et al., 2013 for a review). Thus, future work should investigate how math-anxious individuals make metacognitive control decisions. To investigate this relation, future work could examine whether math anxiety moderates the relation between confidence on a math task and people’s likelihood to ask for help on an item.

Additionally, adults have already completed formal education on fractions, so it is unclear whether the current results would replicate in a sample of children who are just learning about fraction arithmetic. For example, prior work found that children’s math anxiety was related to less accurate retrospective monitoring on an addition and subtraction arithmetic task concurrently and longitudinally in second- to third-graders (Bellon et al., 2021). However, to our knowledge, no prior work has investigated the relation between math anxiety and monitoring accuracy during fraction arithmetic in elementary-aged students. Thus, future work could investigate how math anxiety and monitoring are related in a sample of children still learning about fraction arithmetic.

## Conclusion

The current study evaluated how math-anxious adults monitor their fraction arithmetic ability. We extend previous work on math anxiety and metacognitive monitoring in multiple ways. First, we found that adults were underconfident in their fraction arithmetic performance. Second, our results provide additional support that math-anxious individuals have generally less accurate metacognitive monitoring during an arithmetic task, even when controlling for prior fraction knowledge. Finally, in line with prior work, adults exhibited the postdiction superiority effect (i.e., more accurate retrospective compared to prospective monitoring judgments) when monitoring their fraction arithmetic performance, but math anxiety did not moderate this effect. Thus, adults are better able to retrospectively, compared to prospectively, judge how well they did on fraction arithmetic problems, and even though those with math anxiety may be able to distinguish between problems they solve correctly or not, they may still underestimate their true ability.

### Author’s note

Part of this writing was included as the first author’s master’s thesis. Analyses were pre-registered prior to being conducted but after data were collected (https://osf.io/m4vx8/). All de-identified data and analytic code has been uploaded to osf (osf.io/kfs2h/overview).

## Data Availability

Data and analytic scripts are available on the Open Science Framework (https://osf.io/8c3t6/overview).

## Footnotes

1 Note that we distinguish between math self-concept and math self-efficacy, though these terms are sometimes conflated in prior literature. Math self-efficacy can be defined as a person’s belief in their ability to complete a specific task (Bandura, 1982; Pajares, 1996; Scheibe et al., 2022). Self-efficacy tends to be malleable, future-oriented, and largely focused on one’s perceived confidence. On the contrary, self-concept tends to be relatively stable, past-oriented, and based on one’s perceived competence (Bong and Skaalvik, 2003). Individual instances of a self-efficacy judgment may be based off of one’s self-concept; however, self-concept may reflect an aggregated sense of one’s ability developed through repeated self-efficacy judgments.

2
https://osf.io/m4vx8
