# Supplementary material for: The role of math anxiety in U.S. adults’ ability to metacognitively monitor their fraction arithmetic performance
Source: Front Psychol. 2026 May 7;17:1821523. doi: 10.3389/fpsyg.2026.1821523 (PMC13190410; doi:10.3389/fpsyg.2026.1821523)
Supplement: Supplementary file 1 [file Data_Sheet_1.pdf]

Supplementary Material

Transparency and openness statement

Initially, we planned to test our hypotheses using a single sample. However, we later realized we could analyze the same research questions with a second sample. We first analyzed these samples separately to assess whether the findings replicated, but given the overlap in measures between samples, we report analyses combining both samples to increase statistical power. The samples did not significantly differ in their arithmetic performance, monitoring judgments, or their math self-concept. The samples differed slightly in their number-line estimation performance (i.e., percent absolute error) and their math anxiety. However, both samples completed the same tasks for these measures and we attribute these differences to sampling errors.

Importantly, no results contradict what we report in the main text when we analyze the samples separately. However, some of the results that were non-significant when analyzed as separate samples are significant when analyzed as a combined sample (i.e., postdiction superiority effect on bias). Additionally, math anxiety moderated the postdiction superiority effect in the separated samples but there was no significant interaction in the combined sample. However, the inconsistency is likely due to the effect being small in the split sample analysis (η_p^2's < .03). The larger sample from our combined analysis provides greater statistical power and likely provides a more accurate representation of the relations between math anxiety and monitoring accuracy.

We also made an additional hypothesis in our pre-registration which we no longer report in the main text of our paper. We expected the relation between math anxiety and monitoring accuracy to be attenuated or eliminated when controlling for math self-concept. We chose not to

report analyses controlling for math self-concept due to issues of multicollinearity. Math anxiety and math self concept were highly related, and each had similar relations to our outcome measures.

Comparison of variables between samples

First, we confirmed that both samples performed similarly on the fraction arithmetic task and provided similar aggregate level judgments. T-tests indicated that samples did not differ in arithmetic accuracy, in prospective judgment magnitude, or in retrospective judgment magnitude. However, math anxiety was slightly higher in the first compared to the second sample and number-line estimation (i.e., prior math ability) was less accurate in the first compared to the second sample. Given that math anxiety and prior math ability were measured the same way for both samples, we attribute the difference in math anxiety between samples to sampling error. These small differences in our predictor variables do not affect the interpretation of our results because our combined sample has greater variability and statistical power than either of the individual samples. See Table S1 for comparisons between samples.

Table S1.
Comparison Statistics Between Samples

	Sample 1	Sample 2		
	M (SD)	*M (SD)*	*t* (*df*)	*d*
Fraction arithmetic accuracy	65.58% (28.46%)	69.71% (28.00%)	-1.91 (657)	-0.15
Prospective confidence judgments	54.58% (27.83%)	55.29% (31.71%)	-1.26 (609)	-0.02
Retrospective confidence judgment	56.71% (31.17%)	57.13% (33.29%)	-0.16 (633)	-0.01
Math anxiety	5.63 (2.41)	5.06 (2.73)	2.88 (613)*	0.22*
Math self-concept	3.58 (1.33)	3.71 (1.26)	-1.32 (665)	-0.10
Percent absolute error	13.62% (11.40%)	11.82% (10.65%)	2.13 (668)	0.16*

*$p < .05$

Replication of results with a split sample

First, we report general descriptive statistics and correlations (Tables S2 and S3). Then, we report a hierarchical linear regression in which we predict metacognitive accuracy (as bias or absolute bias) from math anxiety and previous fraction knowledge to evaluate whether math anxiety still predicts monitoring when controlling for prior ability. We then report a series of repeated-measures general linear models in which we predict metacognitive accuracy (as bias or absolute bias) from judgment time (prospective and retrospective). Finally, we report whether math anxiety moderated the effect of judgment time on monitoring accuracy.

As can be seen in Table S2, adults were significantly underconfident in their prospective and retrospective monitoring judgments, inconsistent with our hypotheses and previous work on metacognitive monitoring in math and other domains (Erickson & Heit, 2015; Lingel et al., 2019; Morsanyi et al., 2014). We report inferential statistics comparing monitoring accuracy by time below. Before completing the task, adults predicted on average that they would correctly answer 13 questions in Sample 1 and 13.27 in Sample 2. After completing the task, they reported that they correctly answered 14 questions in Sample 1 and 13.71 questions in Sample 2. However, on average, adults correctly answered 15.7 questions correctly in Sample 1, and 16.7 questions correctly in Sample 2 (out of 24), a level of accuracy greater than they predicted either prospectively or retrospectively.

Table S2.
Descriptive Statistics for Sample 1 and Sample 2

	**Sample 1**	**Sample 2**
**Variables (Possible range)**	**Mean (SD)**	**Mean (SD)**
Fraction arithmetic accuracy (0% to 100%)	65.58% (28.46%)	69.71% (28.00%)
Prospective Confidence Judgment (0% to 100%)	54.58% (27.83%)	55.29% (31.71%)
Retrospective Confidence Judgment (0% to 100%)	56.71% (31.17%)	57.13% (33.29%)
Prospective Bias (-24 to 24)	-2.64 (6.82)*	-3.44 (6.51)*

Retrospective Bias (-24 to 24)	-2.11 (4.92)*	-3.02 (5.18)*
Prospective Absolute Bias (0 to 24)	5.79 (4.45)*	5.65 (4.72)*
Retrospective Absolute Bias (0 to 24)	4.02 (3.53)*	4.40 (4.08)*
Continuous Item-level Gamma (-1 to 1)	0.46 (0.51)*	0.40 (0.53)*
Math Anxiety (0 to 10)	5.63 (2.41)	5.06 (2.73)
Math Self-concept (1 to 6)	3.58 (1.33)	3.71 (1.26)
Percent Absolute Error (0% to 100%)	11.24% (9.32%)	11.82% (10.65%)

* indicates that the mean is significantly different from zero for the measures of monitoring accuracy (i.e., prospective/retrospective bias/absolute bias and dichotomous/continuous gamma). We used a one-tailed test for absolute bias because all values are positive.

Table S3.
Correlation Matrix for Sample 1 (below diagonal) and Sample 2 (above diagonal)

Variable Names	1	2	3	4	5	6	7	8	9	10	11
1. Fraction Arithmetic Accuracy	-	.49**	.77**	-.52**	-.22**	<.01	-.17**	-.12*	-.54**	.57**	-.52**
2. Prospective Confidence Judgment	.49**	-	.72**	.49**	.42**	-.31**	-.36**	-.07	-.54**	.69**	-.37**
3. Retrospective Confidence Judgment	.77**	.72**	-	-.06	.45**	-.14**	-.41**	-.15**	-.59**	.69**	-.48**
4. Prospective Bias	-.52**	.49**	-.06	-	.63**	-.30**	-.19**	.04	<.01	.10*	.16**
5. Retrospective Bias	-.22**	.42**	.45**	.63**	-	-.21**	-.39**	-.06	-.14**	.26**	-.01
6. Prospective Absolute Bias	<.01	-.31**	-.14**	-.30**	-.21**	-	.51**	-.11*	.13**	-.14**	.09
7. Retrospective Absolute Bias	-.17**	-.36**	-.41**	-.19**	-.39**	.51**	-	-.03	.28**	-.21**	.18**
8. Continuous Item-level Gamma	-.12*	-.07	-.15**	.04	-.06	-.11*	-.03	-	<.01	-.07	-.13*
9. Math Anxiety	-.54**	-.54**	-.59**	<.01	-.14**	.13**	.28**	<.01	-	-.71**	.47**
10. Math Self-concept	.57**	.69**	.69**	.10*	.26**	-.14**	-.21**	-.07	-.71**	-	-.45**
11. Percent Absolute Error	-.52**	-.37**	-.48**	.16**	-.01	.09	.18**	-.13*	.47**	-.45**	-

Fraction arithmetic accuracy was reported in Godwin et al., (2023). Prospective = before the task. Retrospective = after the task. Aggregate judgments are judgments of overall performance (e.g., total number correct). Bias reflects the signed difference between judgments and performance; positive bias reflects overconfidence and negative values reflect underconfidence. Absolute bias is the absolute value of the difference between judgments and performance. Gamma is an ordinal measure of relative monitoring accuracy.
*$p < .05$. **$p < .01$.

Is math anxiety related to monitoring accuracy? We expected math anxiety to be related to more accurate prospective monitoring. However, contrary to our hypotheses, math anxiety was not significantly related to prospective bias in Sample 1 ($r < .01$) and related to

greater underconfidence in Sample 2 ($r = -.33$). Additionally, math anxiety was related to less accurate prospective absolute bias in Sample 1 ($r = .13$) and Sample 2 ($r = .28$). In other words, those with more math anxiety were generally less accurate at predicting how many fraction arithmetic items they would solve correctly.

We explored whether math anxiety continued to predict prospective monitoring accuracy after controlling for previous fraction knowledge because the Disruption Account of math anxiety (e.g., Ashcraft & Kirk, 2001) suggests that math ability plays a significant role in the development of math anxiety. As can be seen in Table S4, higher levels of math anxiety continued to significantly predict less accurate prospective monitoring accuracy when controlling for previous fraction knowledge. Thus, math anxiety is still related to less accurate prospective monitoring accuracy even when controlling for prior ability.

We also explored the relation between math anxiety and retrospective aggregate- and item-level monitoring accuracy. Similar to our results on prospective bias, adults with more math anxiety were more underconfident (Sample 1: $r = -.14$; Sample 2: $r = -.30$) and had less accurate absolute bias (Sample 1: $r = .28$; Sample 2: $r = .30$). Although math anxiety was not related to item-level retrospective monitoring accuracy in Sample 1 ($r < .01$), higher levels of math anxiety were related to *more* accurate item-level retrospective monitoring accuracy in Sample 2 ($r = .19$). That is, adults with higher levels of math anxiety were better able to distinguish between items they answered correctly and incorrectly. The positive relation between math anxiety and relative monitoring accuracy is consistent with the hypothesis that math-anxious adults might be more sensitive to errors they make as they solve arithmetic problems. The difference in the relation between math anxiety and item-level relative accuracy and aggregate level prospective and retrospective accuracy suggests that adults likely use different cues when monitoring ongoing

performance compared to making aggregate-level predictions or postdictions. However, the relation between continuous gamma correlations and math anxiety was small and prior work has found no significant relation between math anxiety and item-level monitoring (e.g., Fitzsimmons & Thompson, 2023). Thus, future research should continue to explore the relation between math anxiety and relative monitoring accuracy.

Table S4.
Hierarchical Linear Regression for Math Anxiety Predicting Prospective Monitoring Accuracy

	Prospective Bias (over/under confidence)		Prospective Absolute Bias	
Predictor	b (SE)	β	b (SE)	β
		Sample 1		
Constant	-2.97 (0.87)***	-	4.36 (0.58)***	-
Math Anxiety	-0.30 (0.16)$^{+}$	-0.11	0.21 (0.11)*	0.12*
Prior Fraction Knowledge	0.18 (0.04)***	0.24***	0.02 (0.03)	0.04
	F(2, 376) = 9.50***, R² = .05		*F*(2, 376) = 3.76*, R² = .02	
		Sample 2		
Constant	0.03 (0.73)	-	3.22 (0.56)***	-
Math Anxiety	-1.05 (0.14)***	-0.44***	0.51 (0.10)***	0.30***
Prior Fraction Knowledge	0.16 (0.04)***	0.26***	-0.01 (0.03)	-0.03
	F(2, 302) = 30.15***, R² = .17		*F*(2, 302) = 13.33***, R² = .08	

p< .07 +, p < .05* p < .01 ** p <.001 ***

Is there a postdiction superiority effect? We found evidence of a postdiction superiority effect for absolute bias (i.e., the absolute difference between a judgment and performance) but not bias (i.e., the signed difference between a judgment and performance). Inconsistent with our hypothesis, a repeated-measures general-linear model indicated that bias (i.e., over / under confidence) did not significantly vary by time (i.e., prospectively or retrospectively) for Sample 1, $F(1, 377) = 3.41$, $p = .066$, $\eta_p^2 = 0.01$, or Sample 2, $F(1, 304) = 2.32$, $p = .129$, $\eta_p^2 = 0.01$. However, consistent with our hypothesis, participants' judgments were

more calibrated retrospectively compared to prospectively in both Sample 1, $F(1, 377) = 72.62$, $p < .001$, $\eta_p^2 = 0.16$, and Sample 2, $F(1, 304) = 30.2$, $p < .001$, $\eta_p^2 = 0.09$.

To examine whether the difference in monitoring accuracy by time depended on math anxiety, we added mean-centered math anxiety to the previous models. We found no significant interaction between math anxiety and time for absolute bias for Sample 1, $F(1, 376) = 3.38$, $p = .067$, $\eta_p^2 = .01$, or Sample 2, $F(1, 303) = 0.30$, $p = .587$, $\eta_p^2 < .001$.

However, there was a math anxiety by time interaction when predicting bias for Sample 1, $F(1, 376) = 7.35$, $p < .01$, $\eta_p^2 = 0.02$, and Sample 2, $F(1, 303) = 5.14$, $p = .024$, $\eta_p^2 = 0.02$. As can be seen in Figure S1, adults were less underconfident retrospectively compared to prospectively at low levels of math anxiety in Sample 1 but not Sample 2. A comparison of estimated marginal means at lower levels of math anxiety (-1 SD) revealed a significant difference in bias between retrospective (*EMM* = -1.40, *SE* = 0.35) and prospective (*EMM* = -2.64, *SE* = 0.50) bias in Sample 1, $t(376) = -3.23$, $p = .017$, but not in Sample 2, $t(303) = 0.20$, $p = .996$. Additionally, a comparison of estimated marginal means at higher levels of math anxiety (+1 SD) indicated there was no difference between prospective and retrospective bias for Sample 1, $t(376) = 0.60$, $p = .991$, or Sample 2, $t(303) = -1.08$, $p = .081$ (see Figure S1). Thus, we found evidence of a postdiction superiority effect at low levels of math anxiety in Sample 1, but not Sample 2. We did not find a postdiction superiority effect for people with higher levels of math anxiety. Further, math anxiety was related to more underconfident (i.e., less accurate) judgments retrospectively. These findings are consistent with our interpretation of the Disruption Account of math anxiety: adults with higher math anxiety have disruptions in their working memory that make them less likely to pick up on experience-based cues during problem solving and less likely to update their monitoring judgments after completing the task.

Figure S1.
Postdiction Superiority Effect by High and Low Levels of Math Anxiety in Sample 1 (a) and Sample 2 (b).

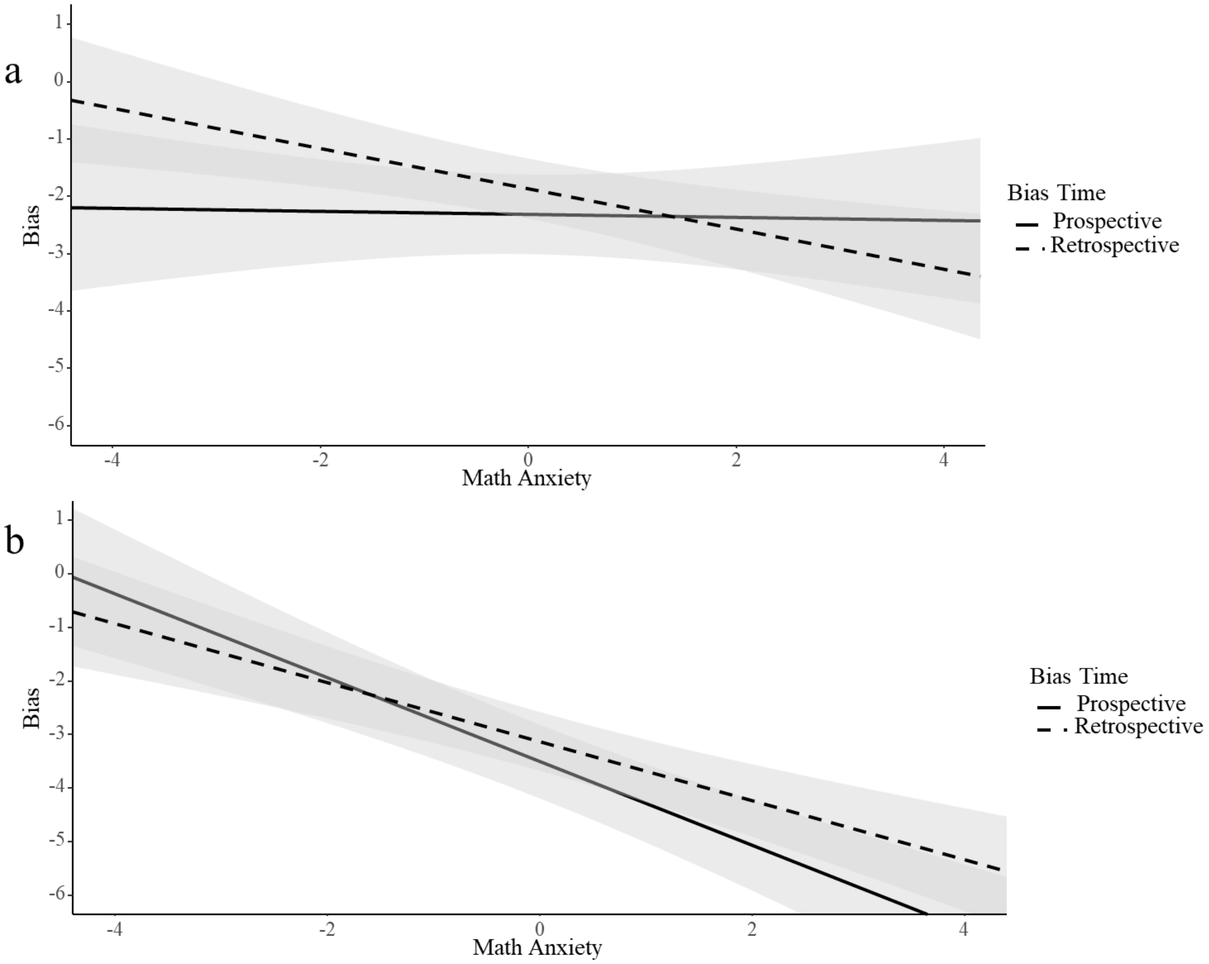

Bias reflects over- and under-confidence for positive and negative values respectively. Sample 1 (a) Sample 2 (b).

Additional preregistered analyses

Although we originally planned to explore whether prior fraction knowledge (as PAE) moderated the relation between math anxiety and prospective monitoring accuracy, we decided that this research exploration was not central to the project's goals and therefore removed it from the main text of the paper. Instead, we report the results of the analysis here.

We found no significant interaction between math anxiety and prior fraction knowledge when predicting prospective bias in the first sample, $\beta = -0.24$, $t(374) = -1.16$, $p = .246$, but there

was a significant interaction in the second sample, β = -0.39, $t(302) = -2.26$, $p = .025$. We did not find any significant interactions between prior fraction knowledge and math anxiety when predicting retrospective bias in the first sample, β = 0.21, $t(374) = 1.01$, $p = .315$, or the second sample, β = -0.30, $t(303) = -1.68$, $p = .095$. We explored the interaction between prior fraction knowledge and math anxiety on prospective bias in the second sample. Simple slope tests indicated that higher math anxiety was related to greater prospective underconfidence when modeled at lower levels of prior fraction knowledge (i.e., higher PAE), $b = -1.41$, 95% CI [-1.82, -1.00]. However, the relation between math anxiety and prospective bias was numerically weaker when modeled at higher levels of prior fraction knowledge (i.e., lower PAE), $b = -0.78$, 95% CI [-1.14, -0.41].

There was also a math anxiety by prior fraction knowledge interaction on prospective absolute bias in the first sample, β = -0.58, $t(375) = 1.18$, $p = .007$, and the second sample, β = -1.02, $t(302) = -5.90$, $p < .001$. We additionally found a math anxiety by prior knowledge interaction for retrospective absolute bias in the first sample, β = -0.64, $t(374) = -3.12$, $p = .002$, and the second sample, β = -0.70, $t(303) = -3.97$, $p < .001$.

To examine the nature of these interactions, we conducted simple slopes tests in which we examined whether the relation between math anxiety and prospective or retrospective absolute bias depended on one's prior fraction knowledge. As shown in Figure 2S, when modeled at higher levels of prior knowledge, higher math anxiety was associated with less accurate judgments in the first and second sample for prospective (Sample 1: $b = 0.46$, 95% CI [0.19, 0.73]; Sample 2: $b = 1.04$, 95% CI [0.77, 1.30] and retrospective (Sample 1: $b = 0.57$, 95% CI [0.36, 0.78]; Sample 2: $b = 0.70$, 95% CI [0.47, 0.93]) absolute bias. However, when modeled at low levels of prior knowledge, there was no significant relation between math

anxiety and absolute bias for the first or second sample for prospective (Sample 1: *b* = -0.12, 95% CI [-0.44, 0.20]; Sample 2: *b* = -0.16, 95% CI [-0.47, 0.13]) or retrospective (Sample 1: *b* = 0.06, 95% CI [-0.18, 0.31]; Sample 2: *b* = 0.06, 95% CI [-0.28, 0.25]) judgments.

Figure S2.
Math Anxiety's Relation to Prospective and Retrospective Absolute Bias at High and Low Levels of Prior Fraction Knowledge in Sample 1 (a) and Sample 2 (b)

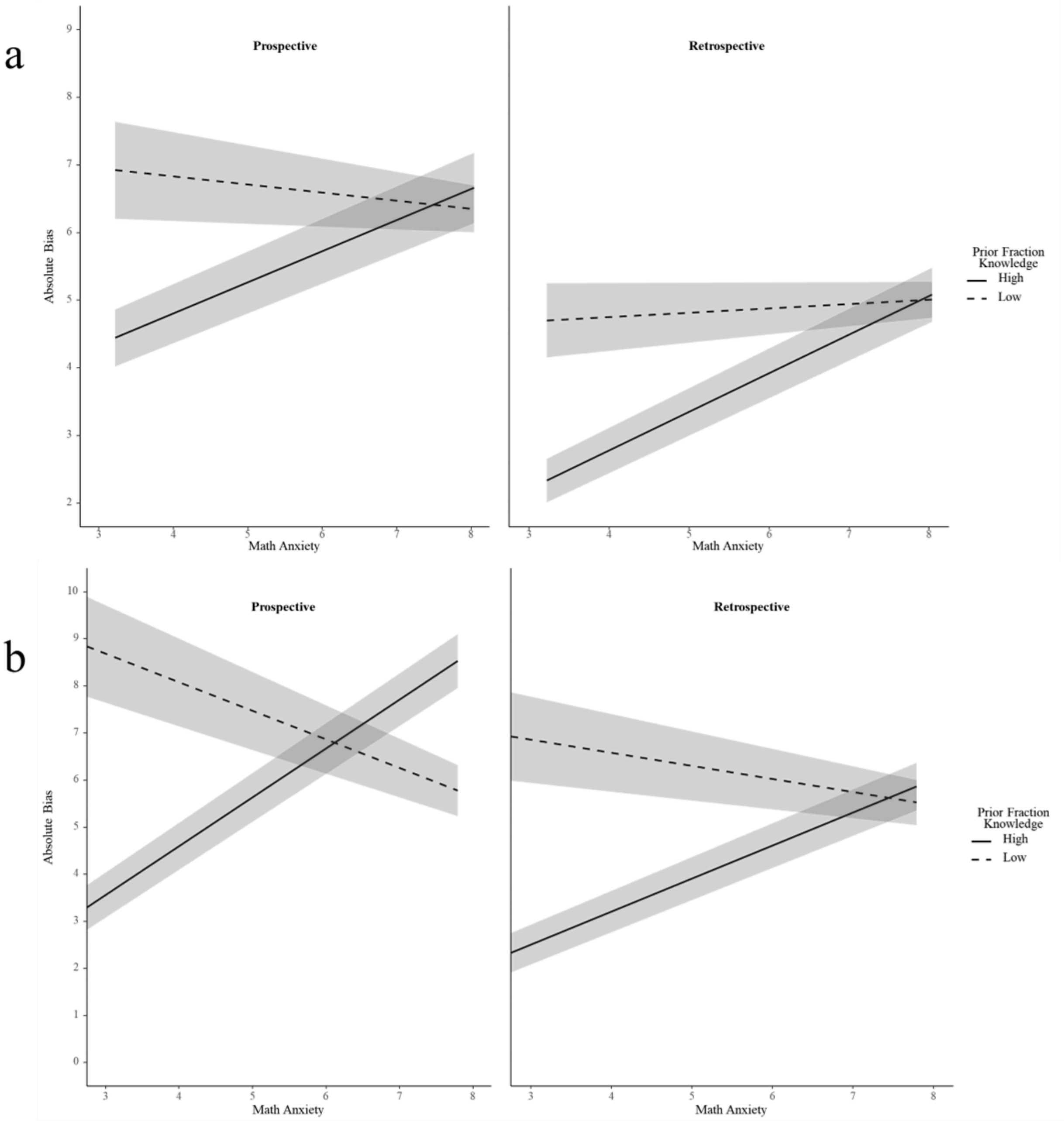

High prior fraction knowledge is low percent absolute error (PAE) while low prior fraction knowledge is high PAE.
Sample 1 (a) Sample 2 (b).

Table S5.
List of Number-line Estimation Stimuli (on a 0-5 scale)

0-5 Estimates
1/19
4/7
7/5
13/9
8/3
11/4
10/3
7/2
17/4
9/2

List of full measures included prior to the manipulation

The following items were measured prior to the manipulation but not included in the current study.

Lay Theories of Experience. Participants completed a 24-item scale, developed by Fisher and Oyserman (2017), to measure how they perceive easy and difficult tasks. There are four subscales in this measure: easy-as-triviality (e.g., "If a task feels easy, my gut says that it doesn't really matter to me"), difficulty-as-importance (e.g., "I know a goal is a key one for me when it feels difficult to work on"), ease-as-possibility (e.g., "When a goal feels easy to attain, then it is probably within my reach"), and difficulty-as-impossibility (e.g., "I know a goal is

impossible for me when it feels difficult to work on") with six items per scale on a 1 (*Strongly disagree*) to 6 (*Strongly agree*).

Subjective Numeracy. Participants completed the eight-item Subjective Numeracy Scale (Fagerlin et al., 2007), to assess their perceived competency to work with numbers on a 1 to 6 scale. See Fagerlin et al. (2007) for the full scale including endpoints.

Math Self-identity. Participants completed a three-item math self-identity task developed by Nosek et al. (2002) to assess whether they consider themselves a 'math person' or an 'arts person' on a scale from 1 (*Strongly disagree*) to 6 (*Strongly agree*).

Math Mindsets. To assess adults' math mindsets, we adapted a mindsets questionnaire from Dweck (1999). Because we are specifically interested in people's mindsets towards math, we included the word "math" before the word "intelligence" in the adapted scale on a six point Likert-like scale from "Strongly agree" to "Strongly disagree". After reverse scoring, higher numbers would indicate higher levels of a growth math mindset while lower numbers would indicate more of a fixed math mindset.

Health Decision-making. Participants completed eight health risk-comprehension problems adapted from Cuite et al., (2008). In this task, participants are informed about the risk of a condition (e.g., "Your risk of cancer E is 1 in 120") and then read that taking a drug or smoking can increase or decrease the risk of the condition (e.g., "but a new drug would cut that risk in half"). After reading the prompt, participants answered what their updated risk would be. Accuracy was operationalized as the number of items correctly answered.

References

Ashcraft, M. H., & Kirk, E. P. (2001). The relationships among working memory, math anxiety, and performance. *Journal of Experimental Psychology: General, 130*(2), 224–237. https://doi.org/10.1037/0096-3445.130.2.224

Cuite, C. L., Weinstein, N. D., Emmons, K., & Colditz, G. (2008). A test of numeric formats for communicating risk probabilities. *Medical decision making : an international journal of the Society for Medical Decision Making, 28*(3), 377–384. https://doi.org/10.1177/0272989X08315246

Dweck, C. S. (1999). *Self-theories: Their role in motivation, personality, and development.* Philadelphia, PA: Psychology Press

Erickson, S., & Heit, E. (2015). Metacognition and confidence: Comparing math to other academic subjects. *Frontiers in Psychology*, *6*. https://doi.org/10.3389/fpsyg.2015.00742

Fagerlin, A., Zikmund-Fisher, B. J., Ubel, P. A., Jankovic, A., Derry, H. A., & Smith, D. M. (2007). Measuring numeracy without a math test: development of the Subjective Numeracy Scale. *Medical decision making : an international journal of the Society for Medical Decision Making, 27*(5), 672–680. https://doi.org/10.1177/0272989X07304449

Fisher, O., & Oyserman, D. (2017). Assessing interpretations of experienced ease and difficulty as motivational constructs. *Motivation Science, 3*(2), 133–163. https://doi.org/10.1037/mot0000055

Fitzsimmons, C. J., & Thompson, C. A. (2023). Why is monitoring accuracy so poor in number line estimation? The importance of valid cues and systematic variability for US College students. *Metacognition and Learning, 19*. https://doi.org/10.1007/s11409-023-09345-y

Godwin, K. E., Thompson, C. A., Kaur, F., Iwai, Y., Fitzsimmons, C. J., & Taber, J. M. (2023). Attending to what's important: What heat maps may reveal about attention, inhibitory control, and fraction arithmetic performance. *Frontiers in Psychology*, *14*. https://www.frontiersin.org/articles/10.3389/fpsyg.2023.1210266

Lingel, K., Lenhart, J., & Schneider, W. (2019). Metacognition in mathematics: Do different metacognitive monitoring measures make a difference? *ZDM*, *51*(4), 587–600. https://doi.org/10.1007/s11858-019-01062-8

Morsanyi, K., Busdraghi, C., & Primi, C. (2014). Mathematical anxiety is linked to reduced cognitive reflection: a potential road from discomfort in the mathematics classroom to susceptibility to biases. *Behavioral and Brain Functions, 10*(1). https://doi.org/10.1186/1744-9081-10-31

Nosek, B. A., Banaji, M. R., & Greenwald, A. G. (2002). Math = male, me = female, therefore math ≠ me. Journal of Personality and Social Psychology, 83(1), 44–59. https://doi.org/10.1037/0022-3514.83.1.44
